# Severity of COVID-19 in hospitalised children in Espírito Santo, Brazil: a comparative analysis of pre- and post-Omicron periods

**DOI:** 10.1590/0074-02760250018

**Published:** 2026-07-10

**Authors:** Brena Ramos Athaydes, Mariane Vedovatti Monfardini, Juliana Santa Ardisson, Gabriela Ferreira Nunes, Rafaela Altoé de Lima, Sabrina Cavalcanti de Barros Fonseca, Rodrigo Pimentel Schade, Teodiano Freire Bastos-Filho, Edson Delatorre, Liliana Cruz Spano, Sandra Ventorin von Zeidler

**Affiliations:** 1 Universidade Federal do Espírito Santo, Centro de Ciências da Saúde, Programa de Pós-Graduação em Biotecnologia, ES, Brasil; 2 Universidade Federal do Espírito Santo, Centro de Ciências da Saúde, Programa de Pós-Graduação em Doenças Infecciosas, ES, Brasil; 3 Hospital Infantil Nossa Senhora da Glória, Vitória, ES, Brasil; 4 Hospital da Polícia Militar do Estado do Espírito Santo, Vitória, ES, Brasil; 5 Hospital Santa Casa de Misericórdia de Vitória, Maternidade ProMatre, Vitória, ES, Brasil; 6 Universidade Federal do Espírito Santo, Centro de Ciências da Saúde, Departamento de Patologia, Laboratório de Genômica e Ecologia Viral, Vitória, ES, Brasil

**Keywords:** paediatrics, viral load, quantitative real-time PCR, phylogenetic analysis, SARS-CoV-2 variants

## Abstract

**BACKGROUND:**

Children experience Coronavirus disease 2019 (COVID-19) often with milder symptoms but severe outcomes.

**OBJECTIVES:**

To compare clinical, epidemiological and viral load profiles of hospitalised paediatric COVID-19 cases before and after the emergence of the Omicron variant in Espírito Santo, Brazil, and to explore factors associated with hospitalisation duration.

**METHODS:**

This cross-sectional observational study included 54 hospitalised children with confirmed severe acute respiratory syndrome coronavirus 2 (SARS-CoV-2) infection, grouped according to epidemiological period: G1 (2020-2021) and G2 (2022). Viral load was quantified by reverse transcription real-time polymerase chain reaction (RT-qPCR), and whole-genome sequencing was performed using Nanopore technology. Group comparisons used chi-square/Fisher's exact tests and Mann-Whitney U tests. Cox regression was applied in an exploratory manner to identify factors associated with hospitalisation duration.

**FINDINGS:**

The median age was 19.5 months and 68.5% were male. Comorbidities were present in 65% of the cohort, with no significant differences between groups. Children in G1 were more often hospitalised for respiratory syndromes, while G2 had a longer intensive care unit (ICU) stay. More than half of the patients required oxygen support, and those needing oxygen at admission were younger. Viral load was higher in G1 than in G2. In the multivariable Cox model, comorbidities [aHR = 0.47; 95% confidence interval (CI): 0.26-0.86] and male sex (aHR = 0.47; 95% CI: 0.25-0.89) were associated with prolonged hospitalisation. Sequencing identified five SARS-CoV-2 lineages (P.1., B.1.1., BA.1*, BQ.1.1, and BE.9), reflecting the transition from pre-Omicron to Omicron periods.

**MAIN CONCLUSIONS:**

The pre-Omicron period was marked by higher viral loads and distinct clinical patterns compared with the Omicron period. Comorbidities and male sex were associated with prolonged hospitalisation. These findings should be interpreted as exploratory and hypothesis-generating given the limited cohort size.

Since the World Health Organisation (WHO) declared Coronavirus disease 2019 (COVID-19) a pandemic, it has become evident that children are affected differently than adults. While most paediatric cases are mild or asymptomatic, some children face severe outcomes, including hospitalisation and, in rare cases, death, with greater severity observed in low- and middle-income countries.^1^ ^,2^

Brazil ranks sixth globally in the total number of confirmed COVID-19 cases.^3^ Over three years (2020-2023), nearly three million children were reported as symptomatic severe acute respiratory syndrome coronavirus 2 (SARS-CoV-2) cases in the country. Of these, almost 60,000 (2.1%) were hospitalised, approximately 14,000 (0.48%) required intensive care unit (ICU) admission, and nearly 5,000 (0.17%) died due to the disease. The southeastern region was the most affected, reporting the highest percentage of hospitalised children compared to other regions. Among the 1.2 million paediatric cases reported in this region, more than 25,000 children (42.8%) were hospitalised, with a case fatality rate (CFR) of 6.4% among hospitalised cases. The State of Espírito Santo stood out with over 130,000 confirmed cases (10.4% of the southeastern total) and a CFR of 17% among hospitalised children, the highest in the region.^4^

The State of Espírito Santo showed a distinct pattern of paediatric COVID-19 outcomes compared with the rest of Brazil. Local surveillance documented sharp increases in paediatric admissions during epidemic peaks, indicating substantial pressure on hospital services.^5^ National analyses also showed unusually high in-hospital mortality among children and adolescents in non-capital municipalities of the state, exceeding 10% and reaching approximately 25% during the early pandemic.^6^ This pattern, typically observed in the most socioeconomically vulnerable regions of Brazil,^7^ highlights the need for a region-specific evaluation of paediatric SARS-CoV-2 infection severity in Espírito Santo.

Although paediatric hospitalisations were less frequent than in adults, severe cases often required intensive care, particularly for conditions like Multisystem Inflammatory Syndrome in Children and Severe Acute Respiratory Syndrome.^8^ ^,9^ Higher viral loads have been strongly associated with worse clinical outcomes and the need for advanced interventions, highlighting the importance of understanding their contribution to disease progression.^10^ ^,11^

Factors such as age, comorbidities, and specific SARS-CoV-2 variants of concern (VOCs) also influence disease severity in children, often leading to ICU admissions and the need for respiratory support.^12^ In Brazil, these variants coincided with increased paediatric hospitalisations, even as adult CFR declined during the Omicron wave, which was marked by high transmissibility and partial vaccine resistance.^13^ ^,14^

As the COVID-19 pandemic subsides, evaluating its impact on paediatric populations remains essential for informing targeted preventive strategies. This cross-sectional study provides an overview of the clinical and epidemiological characteristics of hospitalised children with COVID-19 in Espírito Santo during the pre- (2020-2021) and post-Omicron (2022) periods, with attention to hospitalisation outcomes, including the duration of hospital stay. By integrating genomic, clinical, and epidemiological data, including long-read sequencing to characterise circulating variants and their mutations, we sought to contextualise local paediatric COVID-19 patterns and examine how disease severity, viral load, and healthcare utilisation varied across these periods. Together, these analyses generate region-specific, exploratory evidence that may support public health planning and contribute to broader discussions on paediatric vulnerability in future respiratory epidemics.

## SUBJECTS AND METHODS

*Study population and sample collection* - This cross-sectional observational study enrolled individuals up to 18 years of age diagnosed with COVID-19 and admitted to Nossa Senhora da Glória Children's Hospital, a public referral centre for paediatric respiratory syndromes in Espírito Santo, Brazil. During the pandemic, this hospital was designated as a reference centre for paediatric COVID-19 cases and provided free 24-h emergency care. Most patients were either referred through the state public health regulation system or directly admitted via the hospital's emergency service. The individuals included predominantly resided in Vitória's metropolitan region.

The inclusion criteria were a positive SARS-CoV-2 result confirmed by reverse transcription polymerase chain reaction (RT-PCR) or rapid antigen test. All eligible patients hospitalised in the paediatric pulmonology ward, with or without comorbidities, were invited to participate. A total of 54 patients were divided into two groups based on the sample collection period: Group 1 (G1) included 21 children (from August 2020 to June 2021), while Group 2 (G2) comprised 33 children (from January 2022 to May 2022). Clinical specimens were collected from each participant, including nasopharyngeal/oropharyngeal swabs, saliva (in G2, based on age), and faeces (if available). Samples were transported at 4ºC, processed within 72 h, and stored at -80ºC until testing.

Guardians of children who agreed to participate signed an informed consent form and completed a questionnaire with sociodemographic data, including age (in months) classified in infants (zero-one year), young children (two-five years), older children (six-11 years), and adolescents (12-18 years), sex, place of residence, and prior contact with a suspected case. Additional clinical, symptomatic, and behavioural data were obtained from hospital records and included the presence of comorbidities, symptoms at admission, need for oxygen support, and the type of support used, length of hospital stay, presence of bacterial co-infection, treatments administered, laboratory findings, and clinical outcomes.

*Ethics* - This study was performed in line with the principles of the Declaration of Helsinki. Approval was granted by the Human Research Ethics Committee of the Health Sciences Centre at the Federal University of Espírito Santo (Protocol No. 4.175.329).

*Viral RNA extraction, real-time RT-qPCR, and determination of the viral load* - RNA was extracted using the QIAamp Viral RNA Mini Kit (QIAGEN, Hilden, Germany) following the manufacturer's instructions. SARS-CoV-2 detection employs specific primers and hydrolysis probes targeting two regions of the nucleocapsid gene (N1 and N2) along with an internal control (RNase P-RP), as recommended by the Centres for Disease Control and Prevention (CDC).^15^ Reactions were conducted using a TaqPath™ 1-step RT-qPCR Master Mix (Thermo Fisher Scientific, Waltham, MA, USA) on a StepOnePlus™ PCR system (Applied Biosystems, Carlsbad, CA USA), with negative and positive controls (2019-nCoV, IDT, Iowa, USA) included for assay validation. A positive result was determined by reaching a defined threshold for N1, N2, and RP targets below 0.2, and a cycle threshold (Ct) value below 40.

To quantify the viral load (copy number), the N1 primer/probe set (CDC, IDT) was used for RNA detection. A positive control (IDT) was subjected to serial dilutions (2.0 × 10^0^ to 2.0 × 10^5^ copies/μL) to generate a six-point standard curve, run in triplicate, which was included in every qPCR plate to allow inter-run quantification. Total RNA was quantified in duplicate using the same RT-PCR conditions, and the SARS-CoV-2 viral load was calculated per millilitre of nasopharyngeal/oropharyngeal swabs or saliva sample.

*Library preparation and sequencing (MinION)* - SARS-CoV-2 genome sequencing was conducted in samples with Ct < 25, after cDNA synthesis using the LunaScript™ RT SuperMix (New England Biolabs, MA, USA) and the MIDNIGHT v.4 kit (IDT) [Supplementary data (Table I)]. After amplicon pooling and purification with Ampure XP beads (Nimagen, Nijmegen, Netherlands), library preparation was carried out with the Ligation Sequencing Kit SQK-RBK-110.96, loaded onto Oxford MinION R9.4 flow cells (FLO-MIN106), and then sequenced on the MinION Mk1C device. Raw data were collected using ONT MinKNOW software (Oxford Nanopore Technologies, UK).

*Bioinformatics and phylogenetic analysis* - Quality control and base-calling analysis were performed using Guppy v22.10.7. High-precision database assembly (FASTq files) was executed using the 2019-nCoV-2 Novel Coronavirus Bioinformatics protocol to generate consensus sequences.^16^ All sequences generated in this study were deposited in the GISAID (https://www.gisaid.org)^17^ database [Supplementary data (Table II)], and the other sequences were downloaded from the EpiCoV database in GISAID. Data from this study are available in the GISAID repositories EPI_SET_240816da (https://doi.org/10.55876/gis8.240816da) and EPI_SET_240816th (https://doi.org/10.55876/gis8.240816th).

Multiple sequences alignment of our dataset with the Wuhan reference sequence (NC_045512.2) was performed in MAFFT v7.475^18^ and analysed via maximum likelihood phylogenetic with IQ-TREE v2.1.2^19^ under the GTR+F+R5 nucleotide substitution model, selected by the ModelFinder application.^20^ Branch support was assessed using SH-aLRT and rapid bootstrapping, both with 1,000 replicates. Trees were visualised and edited with FigTree v1.4.4 (http://tree.bio.ed.ac.uk/software/figtree/). Mutational profiles were analysed with Nextclade (https://clades.nextstrain.org) and Outbreak Info (https://outbreak.info), and mutation frequencies were estimated using a personalised Python scripts with Pandas, Seaborn, Matplotlib, and Numpy packages.^21^

*Data processing and statistical analysis* - Descriptive statistics summarised demographic, clinical and epidemiological data, with categorical variables reported as percentages and continuous variables as medians and interquartile ranges (IQR). Group comparisons were performed using Pearson's chi-square test or Fisher's exact test for categorical variables and the Mann-Whitney U test for continuous variables, including viral load measurements. Spearman correlation was used to explore the association between viral load and the time from symptom onset to sample collection.

Cox proportional hazards regression was used in an exploratory manner to access factors associated with hospitalisation duration (time to discharge). All clinically relevant variables were first screened using univariable Cox models, and those with epidemiological relevance to the study question were considered for multivariable adjustment. The final model included year of collection (G1, 2020-2021 vs G2, 2022), sex, comorbidities, and age in months. Year of collection was retained regardless of univariable significance, as comparing clinical outcomes across pandemic periods was a central objective of the study. Age was included as a biological adjustment variable. The proportional hazards assumption was verified using log-log survival plots, with no violations detected. Results are presented as adjusted hazard ratios (aHR) with 95% confidence intervals (95% CI). Statistical significance was set at p < 0.05. The data were analysed using SPSS version 20.0 (IBM, Armonk, New York, USA).

## RESULTS

A total of 65 hospitalised children were recruited for the study, of whom 54 were eligible due to confirmed SARS-CoV-2 infection and were divided into two groups: G1 (n = 21) and G2 (n = 33), representing different epidemiological periods.

The demographic data presented in Table I indicate that most hospitalised children were male, with a median age of 19.5 months (IQR: 6.0-97.75). A higher proportion of children in G1 were aged one-five years, while G2 had a greater percentage of infants younger than one year. Most children lived in urban areas, and one-third in both groups reported prior contact with a suspected case. Fever, runny nose, and cough were the most frequent symptoms. Comorbidities were present in 65% of the cohort, with a numerically higher frequency in G2, although the difference was not statistically significant (p = 0.346). Reported comorbidities included haematological disease (n = 5), solid tumours (n = 3), neurological or genetic conditions (n = 10), respiratory or allergic diseases (n = 7), nutritional issues (n = 6), neonatal complications (n = 5), and other conditions (n = 7), with some children presenting more than on condition.

**TABLE I:** Demographic, epidemiological and clinical features of hospitalised paediatric patients with Coronavirus disease 2019 (COVID-19)

Characteristic, n (%)	Total (n = 54)	G1 (2020-2021) (n = 21)	G2 (2022) (n = 33)	p-value
Age, months (median, IQR)	19.5 (6.0-97.8)	25.0 (5.0-117.5)	12.0 (7.0-88.0)	
Infants (< 12m)	23 (42.6)	6 (28.6)	17 (51.5)	
Toddler and preschool (12m-5y)	14 (25.9)	8 (38.1)	6 (18.2)	
Grade-schooler (6y-11y)	6 (11.1)	3 (14.3)	3 (9.1)	0.950^a^
Adolescent (12y-18y)	11 (20.4)	4 (19.0)	7 (21.2)	0.274^b^
Sex				
Male	37 (68.5)	13 (61.9)	24 (72.7)	
Female	17 (31.5)	8 (38.1)	9 (27.3)	0.404
Place of residence				
Urban	38 (70.4)	16 (76.2)	22 (66.7)	
Rural area	16 (29.6)	5 (23.8)	11 (33.3)	0.455
Contact with a suspected case^c^	18 (33.3)	7 (33.3)	11 (33.3)	0.544
Symptoms before hospitalisation, median (IQR), days	4.0 (1.75-6.0)	3.0 (2.0-6.0)	4.0 (1.0-7.0)	0.694^a^
Clinical features at the presentation				
Fever	41 (75.9)	18 (85.7)	23 (69.7)	0.180
Runny nose	34 (63.0)	14 (66.7)	20 (60.6)	0.653
Cough	41 (75.9)	15 (71.4)	26 (78.8)	0.537
Dyspnea	23 (42.6)	11 (52.4)	12 (36.4)	0.246
Feed refusal	13 (24.1)	9 (42.9)	4 (12.1)	0.010*
Dehydration	5 (9.3)	3 (14.3)	2 (6.1)	0.366^b^
Nausea	6 (11.1)	4 (19.0)	2 (6.1)	0.193^b^
Diarrhea	11 (20.4)	6 (28.6)	5 (15.2)	0.305^b^
Vomiting	9 (16.7)	4 (19.0)	5 (15.2)	0.723^b^
Abdominal pain	6 (11.1)	3 (14.3)	3 (9.1)	0.667^b^
Gastrointestinal symptoms	18 (33.3)	9 (42.9)	9 (27.3)	0.236
Wheezing	16 (29.6)	6 (28.6)	10 (30.3)	0.892
Nasal flaring	10 (18.5)	5 (23.8)	5 (15.2)	0.486^b^
Other symptoms	13 (24.1)	7 (33.3)	6 (18.2)	0.204
Comorbidities^d^	35 (64.8)	12 (57.1)	23 (69.7)	0.346

Data are presented as n (%) or median [interquartile range (IQR)]; percentages of the total were calculated in relation to the group. The Pearson's chi-square test was performed for the p-value. *a*: Mann-Whitney test; *b*: Fisher's exact test; *c*: Some patients presented more than one comorbidity; *d*: Some children were admitted to both the intensive care unit and emergency; HOOD: equipment utilised for administering low-flow oxygen therapy to newborns and infants; TOT: tracheostomy; IOT: orotracheal intubation. *p < 0.05.

Laboratory findings showed thrombocytopenia in approximately 20% of the children, while leukocytosis was more frequent in G2 (p = 0.023); elevated C-reactive protein (CRP) levels were observed in 70.4% of the cohort (Table II). Children with comorbidities had lower platelet (p = 0.007) and leukocyte counts (p = 0.034) compared with those without comorbidities, whereas CRP levels did not differ between groups (p = 0.717).

**TABLE II:** Diagnostic confirmation and laboratory findings of hospitalised paediatric patients with Coronavirus disease 2019 (COVID-19)

Characteristic, n (%)	Total (n = 54)	G1 (2020-2021) (n = 21)	G2 (2022) (n = 33)	p-value
SARS-CoV-2 detection, number tested				
RT-PCR	48 (88.9)	21 (100.0)	27 (81.8)	0.071*b*
Rapid test (Ag)	30 (55.6)	6 (28.6)	24 (72.7)	0.001*
Positive test result				
RT-PCR	36 (66.7)	18 (85.7)	18 (54.5)	
Rapid test (Ag)	12 (22.2)	1 (4.8)	11 (33.3)	
Both	6 (11.1)	2 (9.5)	4 (12.2)	0.016*b
Laboratory findings				
Total leukocyte count (µL), median (IQR)	9630(6057.5-14937.5)	7700 (2595-13500)	10830 (7235-16755)	
Normal (6000-16000 µL)	30 (55.6)	9 (42.9)	21 (63.6)	
Leukocytosis (> 16000 µL)	11 (20.4)	3 (14.3)	8 (24.2)	0.097*a*
Leukopenia (< 16000 µL)	13 (24.1)	9 (42.9)	4 (12.1)	0.023*b
Platelet count (µL), median (IQR)	269000 (198000-374500)	272000 (155000-361500)	266000 (225000-409500)	
Normal (170000-440000 µL)	35 (64.8)	13 (61.9)	22 (66.7)	
Trombocytosis (> 440000 µL)	9 (16.7)	2 (9.5)	7 (21.2)	0.347*a*
Tombocytopenia (< 170000 µL)	10 (18.5)	6 (28.6)	4 (12.1)	0.281*b*
C-reative protein (mg/dL), median (IQR)^c^	0.4 (0.0-88.8)	16.2 (0-46.4)	39.45 (8.9-109.0)	
Normal (≤ 6 mg/dL)	13 (24.1)	6 (28.6)	7 (21.2)	0.082*a*
Elevated (> 6 mg/dL)	38 (70.4)	13 (61.9)	25 (75.8)	0.515*b*

Data are presented as n (%) or median [interquartile range (IQR)]; percentages of the total were calculated in relation to the group. The Pearson's chi-square test was performed for the p-value. *a*: Mann-Whitney test; *b*: Fisher's exact test; *c*: C-reactive protein value was not available for three patients, two in G1 and one patient in G2. SARS-CoV-2: severe acute respiratory syndrome coronavirus 2; RT-PCR: reverse transcription polymerase chain reaction. There was still no rapid test available in G1. *p < 0.05.

During hospitalisation, over half of the children required oxygen therapy, predominantly low-flow ventilation, with a median duration of four days (IQR: 2.75-7.25) (Table III). Patients who required oxygen at admission were younger than those who did not, with median age of 12 months (IQR: 4.0-58.25) vs. 60 months (IQR: 11.5-154.25); p = 0.018.

**TABLE III:** Management and clinical outcomes of hospitalised paediatric patients with Coronavirus disease 2019 (COVID-19)

Management and outcomes, n (%)	Total (n = 54)	G1 (2020-2021) (n = 21)	G2 (2022) (n = 33)	p-value
Oxygen therapy	28 (51.9)	12 (57.1)	16 (48.5)	0.535
Mask/catheter/HOOD	18 (33.3)	10 (47.6)	8 (24.2)	
Days of use (median, IQR)	4.0 (2.75-7.25)	3.5 (2.5-6.5)	4.5 (2.5-10.0)	0.633^a^
Non-invasive ventilation	7 (13.0)	1 (4.8)	6 (18.2)	
Days of use (median, IQR)	4.0 (3.0-35.0)	3.0	9.0 (2.75-39.75)	0.571^a^
TOT/IOT	3 (5.6)	1 (4.8)	2 (6.1)	
Days of use (median, IQR)	4.0 (1.0; 4.0; 5.0)	4.0	3.0 (1.0; 5.0)	1.000^a^
COVID-19 related disease	21 (38.9)	7 (33.3)	14 (42.4)	0.554
Associated bacterial infection	30 (55.6)	10 (47.6)	20 (60.6)	0.349
Pharmacological treatment, n (%)				
Antibiotics	44 (81.5)	18 (85.7)	26 (78.8)	0.723^b^
Oseltamivir	8 (14.8)	3 (14.3)	5 (15.2)	1.000^b^
Corticosteroids	23 (42.6)	14 (66.7)	9 (27.3)	0.004*
Bronchodilator	19 (35.2)	9 (42.9)	10 (30.3)	0.346
Other^c^	36 (66.7)	13 (61.9)	23 (69.7)	0.554
Admitted for respiratory syndrome	30 (55.6)	17 (81.0)	13 (39.4)	0.003*
Length of stay^d^				
Pediatric intensive care unit, n (%)	21 (38.9)	6 (28.6)	15 (45.5)	0.147
Days (median, IQR)	5.0 (4.0-19.5)	4.0 (2.5-5.0)	8.0 (4.0-33.0)	0.029^a^*
Enfermary, n (%)	49 (90.7)	21 (100.0)	28 (84.8)	0.144^b^
Days (median, IQR)	7.0 (4.5-12.5)	7.0 (5.0-18.5)	9.0 (4.0-10.75)	0.278^a^
Outcome, n (%)				
Discharge	52 (96.3)	21 (100.0)	31 (93.9)	0.516^b^
Death	2 (3.7)	0 (0.0)	2 (6.1)	

Data are presented as mean [standard deviation (SD)], n (%), or median [interquartile range (IQR)]; percentages of the total were calculated in relation to the group. The Pearson's chi-square test was performed for the p-value. *a*: Mann-Whitney test; *b*: Fisher's exact test; *c*: medications used to manage comorbidities or complications resulting from hospitalisation; *d*: some children were admitted to both the intensive care unit and emergency, thus being counted in both locations. HOOD: equipment utilised for administering low-flow oxygen therapy to newborns and infants; TOT: tracheostomy; IOT: orotracheal intubation. *p < 0.05.

Reasons for hospitalisation differed between the groups, with over 80% of children in G1 being admitted due to respiratory syndrome, compared to 40% in G2 (p = 0.003). Despite this, G2 presented a longer median ICU stay (p = 0.029). Pneumonia and bacterial infections were the most frequent additional complications. Antibiotic therapy was widely used, and corticosteroid use was more frequent in G1 (p = 0.004) (Table III). The use of salbutamol hydrochloride, a bronchodilator, was notable, with a significant difference in oxygenation methods among children who received this medication (p = 0.004), and the most commonly used method was mask/catheter/HOOD, utilised by 61.1% of these patients (n = 11), data not shown. Two deaths occurred in G2, one associated with pneumonia complicated by bacterial infection, and one in a child diagnosed with Guillain-Barré syndrome, both considered COVID-19-related.

A total of 66 samples (nasopharyngeal/oropharyngeal swabs, saliva, and/or faeces) from 45 children tested positive. Viral load quantification was performed for all samples, but only one sample per patient was included in the analysis (swabs were prioritised, and saliva was used if a swab was unavailable). Positive faecal samples were excluded from viral load analyses (four children had only faecal samples that tested positive), and two samples were below the detection limit. This resulted in 39 samples included in viral load analyses (14 from G1 and 25 from G2).

**Fig. 1:**
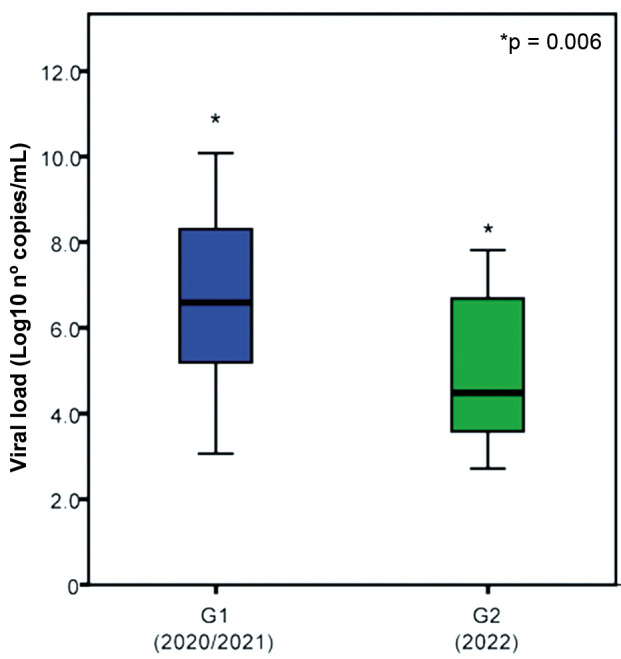
comparison of severe acute respiratory syndrome coronavirus 2 (SARS-CoV-2) viral load between G1 and G2. Viral loads (log10 copies/mL) measured in nasopharyngeal/oropharyngeal swab or saliva samples between groups G1 (2020-2021, blue) and G2 (2022, green). Viral load was significantly higher in G1 compared with G2 (Mann-Whitney U test). *p < 0.05.

Viral load was significantly higher in children admitted during G1 period (2020-2021) compared with G2 (2022) (p = 0.006). Median viral load in G1 was 3.3 × 10^7^ copies/mL (IQR: 1.3 × 10^5^-2.1 × 10^8^), whereas in G2 it was 2.9 × 10^4^ copies/mL (IQR: 3.0 × 10^3^-5.3 × 10^6^) (Fig. 1). When viral load was examined in relation to other baseline characteristics, no significant differences were observed across sex, fever, cough, comorbidities, place of residence or oxygen support at admission (p > 0.05). Viral load was not correlated with the time from symptom onset to sample collection (ρ = -0.20; p = 0.221), and the lack of correlation may represent a potential Type II error (n = 39).

To identify factors associated with hospitalisation duration, univariable Cox regression analyses were conducted. Only sex and comorbidities were significantly associated with time to discharge [Supplementary data (Table III)]. No significant associations were observed for year of collection (G1 vs. G2), age, place of residence, leukocyte count, CRP, oxygen support at admission, viral load, or time from symptom onset to sample collection.

In the multivariable Cox proportional hazards model, comorbidities remained associated with prolonged hospitalisation (aHR = 0.47; 95% CI: 0.26-0.86; p = 0.015). Male sex was also associated with a reduced hazard of discharge (aHR = 0.47; 95% CI: 0.25-0.89; p = 0.020), indicating longer hospitalisation compared with female patients. Year of collection (G1 vs. G2) (aHR = 1.068; 95% CI: 0.60-1.90; p = 0.822) and age in months (aHR = 0.999; 95% CI: 0.99-1.00; p = 0.625) were not associated with time to discharge [Supplementary data (Table IV)].

**Fig. 2:**
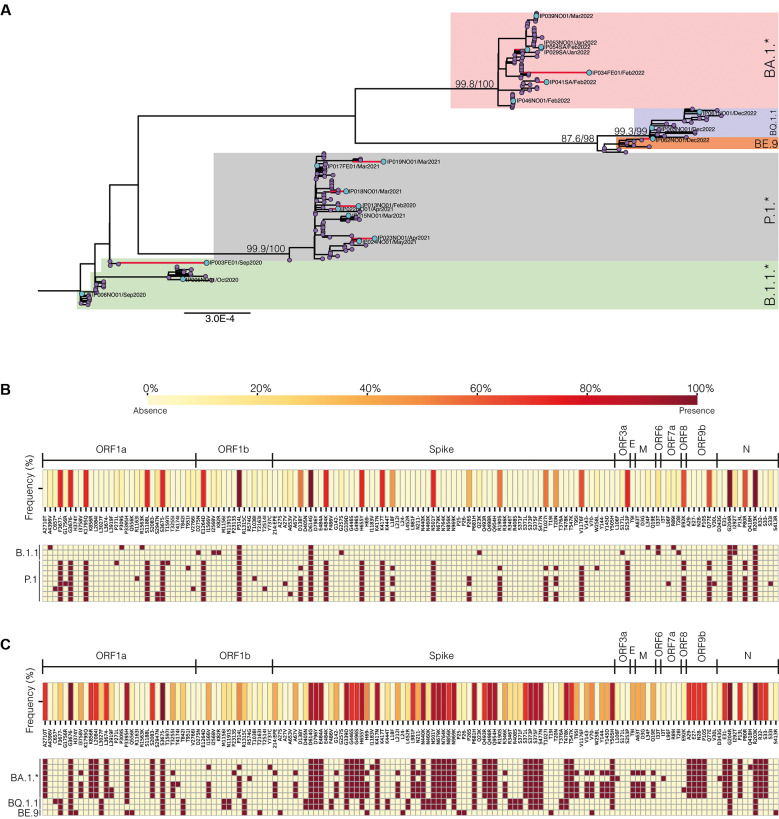
phylogenetic and mutational analysis of severe acute respiratory syndrome coronavirus 2 (SARS-CoV-2) lineages from hospitalised children. (A) Phylogenetic tree rooted with the Wuhan reference strain (NC_045512.2), showing five SARS-CoV-2 lineages: BA.1* (pink), BQ.1.* (blue), BE.9 (orange), P.1.* (purple), and B.1.1.* (green). Red branches and blue circles denote sequences from this study. SH-aLRT/bootstrap values are at key nodes. (B) Heatmap of mutations in the SARS-CoV-2 genome from groups G1 and (C) G2, with colour intensity indicating mutation frequency (%). The light yellow squares represent the absence of mutations, and dark red squares represent the presence of mutations. The upper panels highlight the mutations and their genomic locations identified in this study, not the entire genome.

Only samples with Ct < 25 generated high-quality genomes and were included in the lineage and mutational analyses. We identified five distinct lineages (Nextclade Pango Lineage) among the G1 (n = 11 samples) and G2 (n = 10 samples) SARS-CoV-2 genome sequences: P.1.*, B.1.1.*, BA.1*, BQ.1.1, and BE.9. The observed diversity within the clades revealed specific sublineages: in clade 20B, B.1.1.33 and B.1.1; in clade 20J, P.1 and P.1.14; in clade 21K, BA.1, BA.1.1, BA.1.15, and BA.1.14; in clade 22E, BQ.1.1; and in clade 22B, BE.9 (Fig. 2A).

In G1, mutations were predominantly found in P.1.* lineage, with 26 characteristic and 15 uncommon mutations, and the B.1.1.* lineage, with four characteristic and 14 uncommon mutations. These mutations affect mainly the ORF1a, ORF1b, and Spike regions, with hotspots dispersed across the genome. In G2, the BA.1* lineage presented 67 characteristic and seven uncommon mutations, whereas BQ.1.1 had 39 characteristic and one uncommon mutation. A BE.9 sample presented 17 characteristic mutations and one uncommon mutation. Heat maps indicated a higher mutation density and consistency in G2, particularly in the Spike and ORF1b proteins (Fig. 2B-C).

Among the most represented lineages in both groups, those classified as P.1.* and BA.1* sporadically exhibited uncommon mutations dispersed throughout the genome. However, the *ORF9b:V30L* mutation was found for the first time in P.1.* (https://outbreak.info), whereas the *ORF8:E92K* mutation, which is common at P.1.* lineage, was found in the BA.1* lineage. The *S:ins214EPE* mutation, which is exclusive to the Omicron VOC and used for lineage identification, was detected in samples characterised as BA.1*.^22^

## DISCUSSION

This study provides a detailed characterisation of hospitalised paediatric COVID-19 cases across two epidemiological periods, describing differences in clinical presentation, viral load and circulating SARS-CoV-2 lineages before and after the emergence of the Omicron variant in Espírito Santo, Brazil. Children admitted during the pre-Omicron period showed higher viral loads and were more frequently hospitalised for respiratory syndromes, whereas the Omicron period was characterised by longer ICU stays despite lower viral loads. Comorbidities and male sex were associated with prolonged hospital stay. Genomic sequencing confirmed the transition from P.1.* and B.1.1.* lineages to Omicron sublineages and revealed greater mutational density in G2 genomes, particularly in Spike and ORF1b. These findings outline how viral and clinical characteristics changed across periods and contribute to the understanding of COVID-19 in hospitalised children. Given the modest cohort size, these observations should be regarded as preliminary and interpreted within an exploratory framework.

Consistent with most paediatric COVID-19 studies, our cohort was predominantly male and from urban areas. Fever and cough were the most frequent symptoms, often accompanied by respiratory or gastrointestinal manifestations, in line with reports describing broad but nonspecific symptomatology in young children. Symptom interpretation in children remains challenging due to limited verbal communication, and this contributes to the heterogeneity of clinical presentations observed across paediatric cohorts.^9^

During the pre-Omicron period, most hospitalised children were aged between one and five years and were admitted primarily due to severe acute respiratory syndrome. This observation is consistent with national data showing higher hospitalisation rates and longer lengths of stay among children under five years of age.^23^ One possible contributing factor is the significantly higher viral loads (10 to 100 times greater) in the upper respiratory tracts of children in this age group compared to older children and adults,^24^ which may contribute to increased transmissibility, although this relationship remains complex and multifactorial.^25^ ^,26^

In contrast, hospitalisations in the Omicron period were characterised by a predominance of infants and a numerically higher frequency of comorbidities. Although the overall prevalence of comorbidities did not differ significantly between G1 and G2, clinical outcomes were strongly influenced by underlying conditions. In the multivariable Cox regression model, comorbidities were associated with longer hospitalisation in this cohort, consistent with studies conducted during Omicron predominance showing that chronic diseases substantially increase the risk of severe outcomes and extended hospital stays in children.^27^ ^,28^ Male sex showed a similar association, in line with findings from larger paediatric cohorts describing more severe disease trajectories among boys.^29^

The higher hospitalisation rate among infants during the Omicron wave in our cohort likely reflects a combination of factors. Older children had greater exposure to SARS-CoV-2 in earlier phases of the pandemic and may have developed partial immunity, thereby reducing their risk of hospitalisation. During the Omicron period, a substantial proportion of admissions appeared to be related to incidental SARS-CoV-2 detection or to exacerbations of underlying conditions rather than to primary COVID-19, as routine became widespread and preventive measures were relaxed. Accordingly, admissions in G2 were more frequently associated with complications such as pneumonia, bacterial coinfections, and decompensation of pre-existing comorbidities, whereas admissions in G1 were predominantly related to severe acute respiratory syndrome. These secondary complications were also associated with prolonged hospitalisation after resolution of acute COVID-19 and likely contributed to the high rate of antibiotic use observed in both groups. The association between bacterial infections, antibiotic use, and increased disease severity in COVID-19 patients is well established.^30^ ^,31^

Inflammatory markers further illustrated the complexity of paediatric disease. CRP levels were elevated, reflecting systemic inflammation or concurrent infections, consistent with studies identifying high CRP as a marker of poorer prognosis in children.^32^ ^,33^ Whereas comorbidities were associated with altered leukocyte and platelet counts, they did not influence CRP values, suggesting heterogeneous inflammatory pathways depending on underlying conditions.

Although age was not associated with hospitalisation duration in the multivariable analysis, it played a relevant role in respiratory support requirements. Younger children were more likely to require oxygen therapy at admission, consistent with reports from both pre-Omicron and Omicron waves showing that infants exhibit greater respiratory vulnerability and often require supportive care.^34^ ^,35^ For example, Parri et al.^35^ reported that infants experienced greater disease severity, with most requiring hospitalisation and respiratory support. Fuller et al.^11^ noted higher hospitalisation rates among children aged seven years during the pre-Omicron period (February 2020 to September 2021), with approximately half needing ICU admission and ventilatory support. Similarly, Choi et al.^28^ found increased hospitalisations among young children (zero to four years) with comorbidities during the Omicron period in 2022, often necessitating supplemental oxygen and facing severe disease risks. These findings suggest that, during the Omicron period, disease severity and oxygen needs were more related to comorbidities, bacterial infection control, and hospital complications rather than to intrinsic virological characteristics of the Omicron variant itself.

Many studies have addressed the association between SARS-CoV-2 viral load and disease severity in paediatric populations, with inconsistent results. Elevated viral loads have been reported even in asymptomatic or mildly symptomatic infants,^36^ whereas cohort studies showed no consistent association between viral load and clinical severity, with older children and adolescents often presenting lower viral loads despite symptomatic infection.^37^ ^,38^ In our cohort, viral load was higher in children admitted during the pre-Omicron period than in those hospitalised during the Omicron wave, however, this finding should be interpreted cautiously given the limited sample size.

Taken together, these findings suggest that viral load alone may not fully explain the increased infectivity observed for Omicron variants compared with pre-Omicron strains. Instead, the increased transmissibility of the Omicron variant may be related to its higher affinity for human cell receptors and reduced sensitivity to immune responses from multiple Spike protein mutations.^39^ ^,40^ These mutations are key to viral evolution and influence both transmissibility and disease severity. We identified several significant mutations across SARS-CoV-2 lineages, revealing distinct public health and clinical implications, consistent with previous studies.^40^ ^,41^ G2 samples presented a higher number of mutations and a greater density of mutation hotspots compared to G1, particularly in the Spike protein, where Omicron variants showed significantly more mutations than earlier variants.

The *S:D614G* mutation, identified across various SARS-CoV-2 lineages, is associated with lower RT-PCR Cts, indicating higher viral loads and increased infectiousness.^42^ ^,43^ Other frequently observed mutations in our study, such as *S:V1176F* (common in P.1.*), *N:P13L* (common in BA.1*), *N:R203K* (observed in all lineages), *N:G204R* (common in all lineages), and *ORF3a:S253P* (common in P.1.*) are associated with severe outcomes and increased hospitalisations.^44^ Additionally, the *N:R203K+G204R* nucleocapsid mutation, prevalent in all our samples, has been associated with increased infectivity.^45^ ^,46^ The Omicron subvariant BQ.1.1 exhibited mutations that confer increased resistance to antibody neutralisation, primarily driven by the *S:N460K*, *S:K444T*, and *S:R346T* mutations.^14^ These mutations may enhance interactions with the ACE2 receptor, intensifying cell-to-cell fusion and thus boosting resistance to neutralisation, along with evading therapeutic monoclonal antibodies.^47^ ^,48^

Nagy et al.^44^ highlighted other mutations associated with severe outcomes, suggesting potential impacts on the virulence or immune evasion. One such mutation is *N:I292T*, which was also found in our study among G1 children infected with B.1.1.* lineage. The mutations *T19I*, *A27S*, *S371F*, *S373P*, *S375F*, *T376A*, *D405N*, *R408S*, *G339D*, *K417N*, *N440K*, *S477N*, *T478K*, *E484A*, *Q493R*, *Q498R*, *N501Y*, *Y505H*, *D614G*, *H655Y*, *N679K*, and *P681H* that we observed have also been previously associated with potential viral adaptation for increased infectivity in children. The transmembrane mutation *E:T9I* was also identified, though its role in transmission dynamics and COVID-19 severity is not yet clear.^49^

An intriguing question arises regarding potential differences in the incidence of SARS-CoV-2 Omicron variants between adults and children, given the divergent clinical manifestations. Despite no significant genomic differences across age groups, the rise in paediatric cases suggests unique pathogenic characteristics of Omicron variant, potentially linked to altered Transmembrane Serine Protease 2 (TMPRSS2) expression, reduced S protein cleavage efficiency, and a high incidence of C to T nucleotide mutations in the variant's genome.^49^

This study has several limitations. The small sample size, which reflects both the low number of hospitalised children during the pandemic and challenges in sample acquisition, reduced the statistical power to detect subtle associations, limited the number of covariates that could be included in multivariable models, and prevented robust subgroup analyses. Consequently, the statistical findings should be interpreted as exploratory and hypothesis-generating rather than definitive. Generalisability is limited by the single-centre design, although the hospital served as the official paediatric COVID-19 referral centre for the State of Espírito Santo, supporting the regional representativeness of the cohort. Selection bias may also be present, as the absence of a comparison group of non-hospitalised children and the exclusion of antigen-only cases likely reduced the representation of milder infections. In the genomic analysis, sequencing was restricted to samples with Ct < 25, introducing a selection bias toward infections with higher viral loads, driven by technical requirements for high-quality genome recovery rather than analytical choice. As a result, the mutational profiles and lineage distributions reported here reflect only this subgroup and may overestimate variants enriched under high viral burden, limiting inference regarding low-viral-load infections or their clinical behaviour. Vaccination did not influence our findings, as paediatric immunisation had not yet begun during the study period.

*In conclusion* - This study suggests that the emergence of the Omicron variant was associated with changes in the clinical and epidemiological profile of hospitalised children, while differences in viral load alone did not explain disease severity or hospitalisation duration. Host-related factors, particularly comorbidities and sex, played an important role in prolonged hospital stays, underscoring their relevance in paediatric COVID-19 outcomes. Genomic analyses documented the transition from pre-Omicron to Omicron lineages and described the accumulation of mutations associated with viral adaptation and transmissibility. Considering the exploratory nature of the present cohort, these findings contribute to the understanding of paediatric COVID-19 across distinct epidemiological periods and support the integration of clinical and genomic surveillance to inform patient management and public and regional health strategies.

## SUPPLEMENTARY MATERIALS

Supplementary material

## Data Availability

The datasets generated and analysed during the current study are available in the EPI_SET_240816da repository (https://doi.org/10.55876/gis8.240816da) and EPI_SET_240816th repository (https://doi.org/10.55876/gis8.240816th) from the GISAID database. All datasets on which the conclusions of the paper rely are going to be made available post-publication to readers via community-accepted repositories linked to the manuscript via permanent identifiers.
