# Supplementary material for: Severity of COVID-19 in hospitalised children in Espírito Santo, Brazil: a comparative analysis of pre- and post-Omicron periods
Source: Mem Inst Oswaldo Cruz. 2026 Jul 10;121:e250018. doi: 10.1590/0074-02760250018 (PMC13363245; doi:10.1590/0074-02760250018)
Supplement: Supplementary material [file 1678-8060-mioc-121-e250018-s1.pdf]

TABLE I

Oligonucleotide sequences used in the cDNA amplification process

poolA_1200_23_Left_Omicron	ACTTTAGAGTTCAACCAACAGAATCT
poolA_1200_21_Right_Omicron	GTGTATGATTGAGTTCTGGTTGTAAG
poolB_1200_22_Right_Omicron	AACAGATGCAAATTTGGTGGCG
poolB_1200_24_Left_Omicron	GCTGAATATGTCAACAACTCATATGA
poolB_1200_28_Left_Omicron	TTTGTGCTTTTTAGCCTTTCTGTT

TABLE II

All sequences generated in this study and deposited into the GISAID database

Strain_ID	Clade	Nextclade_pango	Collection_date
hCoV-19/Brazil/ES-LabVig-IP003/2020	20B	B.1.1.33	sep/20
hCoV-19/ Brazil /ES-LabVig-IP005/2020	20B	B.1.1.33	oct/20
hCoV-19/ Brazil /ES-LabVig-IP006/2020	20B	B.1.1	sep/20
hCoV-19/ Brazil /ES-LabVig-IP013/2020	20J	P.1	feb/21
hCoV-19/ Brazil /ES-LabVig-IP015/2021	20J	P.1	mar/21
hCoV-19/ Brazil /ES-LabVig-IP017/2021	20J	P.1	mar/21
hCoV-19/ Brazil /ES-LabVig-IP018/2021	20J	P.1	mar/21
hCoV-19/ Brazil /ES-LabVig-IP019/2021	20J	P.1	mar/21
hCoV-19/ Brazil /ES-LabVig-IP022/2021	20J	P.1	apr/21
hCoV-19/ Brazil /ES-LabVig-IP023/2021	20J	P.1.14	apr/21
hCoV-19/ Brazil /ES-LabVig-IP024/2021	20J	P.1.14	may/21
hCoV-19/ Brazil /ES-LabVig-IP029/2022	21K	BA.1	jan/22
hCoV-19/ Brazil /ES-LabVig-IP034/2022	21K	BA.1	feb/22
hCoV-19/ Brazil /ES-LabVig-IP039/2022	21K	BA.1.1	jan/22
hCoV-19/ Brazil /ES-LabVig-IP041/2022	21K	BA.1.15	feb/22
hCoV-19/ Brazil /ES-LabVig-IP046/2022	21K	BA.1.14	feb/22
hCoV-19/ Brazil /ES-LabVig-IP053/2022	21K	BA.1.1	jan/22
hCoV-19/ Brazil /ES-LabVig-IP054/2022	21K	BA.1.1	feb/22
hCoV-19/ Brazil /ES-LabVig-IP061/2022	22E	BQ.1.1	dec/22
hCoV-19/ Brazil /ES-LabVig-IP063/2022	22E	BQ.1.1	dec/22
hCoV-19/ Brazil /ES-LabVig-IP062/2022	22B	BE.9	dec/22

TABLE III

Univariable Cox proportional hazards analysis of factors associated with time to discharge among paediatric patients hospitalised with Coronavirus disease 2019 (COVID-19)

Variable	B	SE	Wald	df	p-value	HR (ExpB)	95% CI for HR
Group G1 (2020/2021)	0.103	0.288	0.127	1	0.722	1.108	0.630-1.950
Male sex	-0.917	0.317	8.399	1	0.004*	0.400	0.215-0.743
Age (months)	-0.001	0.002	0.237	1	0.626	0.999	0.995-1.003
Urban residence	-0.437	0.312	1.960	1	0.162	0.646	0.350-1.191
Leukocyte count (cells/µL)	0.000	0.000	0.004	1	0.949	1.000	1.000-1.000
C-reactive protein (mg/dL)	-0.001	0.003	0.178	1	0.673	0.999	0.992-1.005
Oxygen support at admission	-0.260	0.284	0.837	1	0.360	0.771	0.442-1.346
Comorbidities	-0.885	0.300	8.683	1	0.003*	0.413	0.229-0.743
Viral load (log_{10})	-0.045	0.080	0.317	1	0.573	0.956	0.816-1.119
Time from symptom onset to sample collection (days)	-0.023	0.018	1.606	1	0.205	0.977	0.943-1.013

B: regression coefficient; SE: standard error; HR: hazard ratio; CI: confidence interval; HR < 1 indicates slower discharge (longer hospitalisation). Reference categories: G2 group (2022), female sex, rural residence, no oxygen support at admission, and absence of comorbidities. Continuous variables were analysed on their original scale. *p-value < 0.05.

TABLE IV

Multivariable Cox proportional hazards model for time to discharge among paediatric patients hospitalised with Coronavirus disease 2019 (COVID-19)

Variable	B	SE	Wald	df	p-value	aHR (ExpB)	95% CI for aHR
Group G1 (2020/2021)	0.066	0.295	0.050	1	0.822	1.068	0.600-1.904
Male sex	-0.758	0.325	5.441	1	0.020*	0.468	0.248-0.886
Comorbidities	-0.750	0.308	5.922	1	0.015*	0.473	0.258-0.864
Age (months)	-0.001	0.002	0.239	1	0.625	0.999	0.994-1.003

B: regression coefficient; SE: standard error; aHR: adjusted hazard ratio; CI: confidence interval; aHR < 1 indicates slower discharge (prolonged hospitalisation). Reference categories: G2 group (2022), female sex, and absence of comorbidities. *p- value < 0.05.

DATA AVAILABILITY

GISAID Identifier: EPI_SET_240816da
doi: 10.55876/gis8.240816da

All genome sequences and associated metadata in this dataset are published in GISAID's EpiCoV database. To view the contributors of each individual sequence with details such as accession number, Virus name, Collection date, Originating Lab and Submitting Lab and the list of Authors, visit 10.55876/gis8.240816da

Data Snapshot

- EPI_SET_240816da is composed of 21 individual genome sequences.
- The collection dates range from 2020-09 to 2020-09;
- Data were collected in 1 countries and territories;
- All sequences in this dataset are compared relative to hCoV-19/Wuhan/WIV04/2019 (WIV04), the official reference sequence employed by GISAID (EPI_ISL_402124). Learn more at https://gisaid.org/WIV04.

DATA AVAILABILITY

GISAID Identifier: EPI_SET_240816th
doi: 10.55876/gis8.240816th

All genome sequences and associated metadata in this dataset are published in GISAID's EpiCoV database. To view the contributors of each individual sequence with details such as accession number, Virus name, Collection date, Originating Lab and Submitting Lab and the list of Authors, visit 10.55876/gis8.240816th

Data Snapshot

- EPI_SET_240816th is composed of 194 individual genome sequences.
- The collection dates range from 2020-03-06 to 2023-03-01;
- Data were collected in 21 countries and territories;
- All sequences in this dataset are compared relative to hCoV-19/Wuhan/WIV04/2019 (WIV04), the official reference sequence employed by GISAID (EPI_ISL_402124). Learn more at https://gisaid.org/WIV04.
